# Employee participation in achieving industrial safety and health – Vision 2020

**DOI:** 10.4103/0019-5278.55120

**Published:** 2009-08

**Authors:** H. Mahadevan

**Affiliations:** Dy. General Secretary, All India Trade Congress (AITUC), New Delhi - 110001, India E-mail: aitucong@bol.net.in

## INTRODUCTION

Mr. Kofi Annan, former Secretary General of the United Nation said “all too often lives are shattered unnecessarily of poor working conditions and inadequate safety systems. Let me encourage everyone to join the ILO in promoting safety and health at work. It is not only sound economic policy; it is basic human right”.

Safety and health is not just for specialists and professionals. It should become the concern of all people at workplaces. We have to collectively develop the conscience of the common people. In short, the worker should return home in the same condition as s/he came to work. This shall be our vision now as well as in 2020.

More than 100 years ago it was said “we have nothing to lose but our chains.” Now the chains are, of course, not in the hands but in brains. Let us break these chains and guarantee a safe and healthy life to the working masses as well as their bosses. In the name of facing global competition, cost cutting, quicker output, better profits etc. no one is permitted either by law or by ethic to cause any hazard in the work place and surroundings - biological hazard or muscular skeletal disease etc. Our future vision is certainly an inclusive one. Employee participation is naturally inevitable in this process.

## NATIONAL PERSPECTIVE

In the current decade, a national policy, system, program and profile are needed.. Ethical criteria and legal obligations are well within this frame work.

Workers in many countries still face the grimmest threat to their occupational safety and health. There are similarities - be it a factory fire at ‘Triangle Shirtwaist factory’ in New York in 1911 killing 146 workers or 82 years later, on May 10 1993, at Kedar toy factory in Thailand. Silicosis, believed to have been found in workers who built the Pyramids in Egypt continues to be a dreadful disease even now. The two notorious disasters – Three Mile Island of Pennsylvania in 1979, Chernobyl in Ukraine in 1986 still warn of future nuclear plant accidents. Since the first documented case of carcinogens and sectoral cancer in the London Child – Chimney – Sweepers, comparable occupations causing environmental cancer exist even in 2008. The giant multi national corporation (MNC) – Union Carbide Factory disaster at Bhopal, whose sufferers continue in the next century, did not provide enough lessons to prevent subsequent chemical disasters.

### 

#### What do all these illustrations drive us to?

The dismal record of ratifications and implementations of OSH conventions and recommendations, manufacture and usage of even banned chemicals, work related stress and hazards reducing the active life of the ‘educated bonded labor’ in the sunrise industry, IT sector, reproductive hazards, the untold miseries silently suffered by vulnerable workers in the growing informal sector, an aftermath of globalization, and similar horrifying experiences drive us to the conclusion that we have not done enough and the intellectual jugglery to justify what has been unfortunately happening cannot continue any more. Worker-participation is a pre-requisite to achieve industrial safety and health

### Certain Principles AITUC Upholds

- We reaffirm the precedence of the human being over the economy. Marketization of trade, particularly traded values, has been extended to all possible fields; marketization of human work is one of the most shocking components. One of the services which is present particularly in GATS is another extremely working aspect.
- Investment in safety and health at work is considered a ‘cost’ in many cases, assessed from the viewpoint of a relation with benefits and productivity. This economic and financial analysis generally leaves ethical criteria and legal obligations completely aside.
- Strengthening safety and health at work implies that efficient and accessible services are made available to the workers. The privatization of public health services and reduction of the budget for ministry of labor and within the context for the labor inspectorate services have been conducive to weakening these services.
- Safety and health at work is essential to guarantee ‘decent work’. In our opinion, decent work must be based on dignity of the workers. Respect and dignity of workers and of every human being is neither negotiable nor of financial value.
- The field of safety and health at work is extremely technical. The reflection and progress in this field will make it possible to ensure better working conditions for workers throughout. That debate includes political stakes.
- In recent years, much to our concern, many employees tend to advocate an alleviated, less independent control system which develops no jurisprudence, a debate including the will to lower the denunciation criteria and to revise downward the existing standards without challenging the need for adjustment.
- ILO standards must effectively become the basis of an undeniable super-rational social legislation whose evolution must be managed in a tripartite manner. Like any other provision of the law, the adoption of a standard satisfies the need for regulating a given situation.

## RATIFICATION OF INTERNATIONAL INSTRUMENTS AND SETTING STANDARDS

It shall be our sincere and serious endeavor to see it that our national government ratifies all the safety and health conventions and accompanying recommendations and frames/amends/updates our laws accordingly. This shall form part of our vision.

### Convention No. 155, Occupational Safety and Health 1981

The aim of the convention is a coherent policy on occupational safety, occupational health and working environment and to ensure communication and co-operation at all levels in this area. It is the most important ILO standard on safety and health it applies to all branches of economic activities and to all workers.

The government should, in consultation with representative organizations of employers and workers, formulate, implant and periodically review a national policy on occupational safety, health and the working environment. The aim of the policy is to prevent accidents and injury to health from work by minimizing so far as it is reasonably practicable the causes of hazards inherent in the working environment. The convention defines the main sphere of action of such a policy. It lays down a series of detailed provisions concerning action at the national level of the undertaking. However, this convention is not yet ratified by India.

### Convention 161, Occupational Health Services Convention and Recommendations, 1985

This convention is also important, but neglected. The important points are: inspectors are supposed to interact with the workers organizations and bring out annual reports but both these do not happen in the absence of the ratification of the convention.

### Convention No. 170, Chemicals

The aim of the standard is to protect workers from the hazards of the chemicals, prevent or reduce the incidence of chemically induced illness and injuries resulting from the use of chemicals at work and consequently enhance protection of the general public and environment. It applies to all branches of economic activities in which chemicals are used. India has not ratified this convention. However, 1987 amendment to Factories Act does require employers to provide workers with information along with the lines required in the convention.

### Convention No. 174. Prevention of Major Industrial Accidents

The aim of the convention is to prevent major accidents involving hazardous substances and limit consequences of such accidents. The importance of this convention hardly needs spelling out. The Bhopal disaster which caused the death of 13,000 people was one of the major reasons for this convention.

Since Bhopal there have been several other accidents which led to casualties - Nagothane in 1990 (25 dead) and Panipat Natural Fertilizers plant in 1992 (10 dead). Nine workers were killed in sleep by a toxic gas release from Century Rayons Plant at Shahad, Maharashtra. There were accidents at Vizag, Agra shoe factory and in Amritsar followed by a major accident in Delhi.

### Convention No. 167, Safety and Health in Construction 1988

A large number of workers are employed in the construction industry in India. Existing standards of construction need to be improved. Health and safety are important aspects. However, India has not ratified the convention.

### Convention on Health and Safety in Agriculture at ILO Conference, May, 2000

ILO conventions and recommendations on safety, occupational health and work environment will have to be ratified by the Government. of India and our existing laws to be attuned to the provisions contained in these conventions, besides enacting laws, wherever they do not exist, for example, agriculture and implement them without relaxation or dilutions.

### Directive Principle on OSH Related Matters and Our Responsibility

A few years ago the Supreme Court of India has turned the spotlight on one issue contained in Article 44 of the Constitution of India “Directive principles of state Policy” and regretted that the Indian Parliament has not given effect to Article 44 of the Constitution, though it directs non-bindingly. The same Article “directs” to:

- secure the health and strength of workers, men and women
- act against abuse of children/child labor
- provide just and humane conditions of work
- secure the participation of workers in the management etc.

In the light of the above direction of the Supreme Court on one such provision of Article 44, it is incumbent on the Government to enact laws on the other most important provisions also including OSH. It shall be our responsibility to campaign towards realizing the principles enshrined in Article 44 of our Constitution to benefit the working people and in turn the industry and productivity which ultimately is in the best interest of the national economy. How can this escape our vision?

## UNORGANIZED / INFORMAL SECTOR-OSHMS NEEDS

On “giving priority to the unorganized sector” the (first) National Commission on Labor has observed “that malpractices prevail in unorganized sector and small scale industries where the arm of the law does not reach and where workers have little awareness of their rights and also where payment is on the basis of piece rates and there is no guarantee that work is properly measured/weighed. It was further observed that the implementing machinery should be more vigilant in cases of units where malpractices are likely to be common. In small establishments and traditional industries, malpractices could be cancerous. More drastic remedies are called for in such cases. Emphasis on implementation of labor laws in unorganized sector has to become a declared statutory policy of the country.

### Special OSHMS Needs of Unorganized Sector

- Unorganized sector employs about 90% of the workforce
- Seven major sectors identified by working group (Tenth Plan) are agriculture, construction, shops and establishment, bidi and cigar manufacturing, home work, eating places and waste management
- Very few legislative measures exist
- Employment of vulnerable group of workers like women and children is much more common
- Many hazardous processes are being carried out
  The organized workers unions/representatives have an important and positive role to play with regard to their un informed brothers and sisters and thus extend their organic solidarity to the unorganized/informal sector. This shall continue to be our agenda towards achieving safety and health.
- AITUC's Campaign / Action Plan to Achieve Vision

### AITUC's Campaign / Action plan includes

- Formulation of National policy on OSHE
- Constitution of a National Tripartite Commission on OSHE
- Codification of OSHE legislations into one umbrella Act
- Empowerment of tripartite machinery at all levels to monitor /review implementation
- Training Program for Trainers
- Adoption of trade Unions own policy on OSHE and directing for implementation at all levels
- Bringing out simple literature / training material
- Participation in forums such as pollution control boards (PCB)
- Joint campaign against manufacturing of banned chemicals, toxic wastes etc.
- Code for MNCs
- Taking up the cause of workers in unorganized / informal sector.
- Inclusion of safety, health and environmental demands as “new collective bargaining agenda” in the charter of demands ands to ensure agreements contain ‘Green clauses’ etc.

## AITUC CONVICTION

We are of the firm conviction that SAFETY AND HEALTH MUST BE A FUNDAMENTAL HUMAN RIGHT OF THE WORKERS. A decent work agenda of the ILO includes safe working, without occupational diseases and accidents. This cannot be achieved without asserting safety and health as a fundamental right of workers, irrespective of relations and economic condition. It is necessary to remove the wrong belief that many occupational diseases are necessary components and inevitable constituents of their work; that any solutions to these problems would entail high degree of technical expertise and large financial investment. This negative thinking and unhealthy attribution should be proved wrong and be totally altered by means of appropriate training on OSHE and education at all levels. AITUC's goal is to make a different industrially advancing nation which is accident free, occupational disease free, pollution free and polluters free in the era of implementing decent work agenda.

